# The role of the internet of things in avoiding the road traffic crashes 

**Published:** 2019-08

**Authors:** Alireza Razzaghi

**Affiliations:** ^*a*^Safety Promotion and Injury Prevention Research Center, Shahid Beheshti University of Medical Sciences, Tehran, Iran.

## Abstract

**Background:**

Road traffic injuries (RTIs) are one of the main health-related problems around the world. The ‎Internet of Things (IoT) is an internet platform and novel paradigm which has a great prospect ‎in health systems. There are different aspects of road safety which IoT can be used to avoid ‎crashes. This study aimed to investigate the role of the internet of things in road safety and ‎avoiding road traffic crashes. ‎

**Methods:**

This is a review study, which was done in the related studies published from 2000 to 2019. The ‎role of IoT has investigated in different aspects of road, vehicle, road user behavior, ‎environment, and weather.‎

**Results:**

There is an increasing trend in the using of IoT in different aspects of road safety especially in ‎the production of modern vehicles. By using a properly IoT management system, equipment ‎and vehicle operators are less likely to encounter dangerous road conditions. Modern vehicles ‎are equipped with some advanced electronic systems such as; driver assistant system, distance ‎sensing, detection of improper driving, active safety system, adaptive cruise, electronic stability ‎control, lane departure warning and lane keeping that aid a vehicle driver while driving. ‎Moreover, the IoT has a remarkable role in prediction and alarming the weather condition and ‎assessing the environment to avoiding the road traffic crashes. ‎

**Conclusions:**

The technology of IoT is a novel and cost-effectiveness way that can prevent road crashes. As ‎the RTIs grow around the world, the IoT technology can reduce the number of crashes on the ‎road. It is essential to pay more attention by governments to applying the IoT in the right way to ‎promote road safety and reducing road traffic injuries.‎

**Keywords:**

Internet of things, Road traffic crashes, Injuries

